# PREDICTIVE FACTORS FOR COMPLETE AND INCOMPLETE EVALUATION OF SMALL INTESTINE BY ENDOSCOPIC CAPSULE

**DOI:** 10.1590/0102-672020200002e1532

**Published:** 2020-11-20

**Authors:** Andréia Sopran SCOPEL, Fernando Issamu TABUSHI, Luis Fernando Kubrusly, Paula Bechara POLETTI, Artur Adolfo PARADA, Milena Perez MOREIRA, Thiago Festa SECCHI

**Affiliations:** 1 Postgraduate Program in Principles of Surgery, Mackenzie Evangelical Faculty of Paraná/Medical Research Institute, Curitiba, PR, Brazil; 2 Gastrointestinal Endoscopy Service, 9 of July Hospital, São Paulo, SP, Brazil

**Keywords:** Capsule endoscopy, Small intestine, Crohn disease, Comorbidity, Cápsula endoscópica, Intestino delgado, Doença de Crohn, Comorbidade

## Abstract

**Background:**

The small-bowel is the most difficult segment to be visualized by traditional endoscopic methods. The need for its exploration led to the development of capsule endoscopy. The percentage of the complete examination varies and still remains uncertain the factors that influence the complete and incomplete examination.

**Aim:**

Evaluate the factors that interfere with the completeness of the endoscopic evaluation by the capsule.

**Methods:**

A prospective study in which were included 939 patients divided into two groups: complete group (CG) and incomplete group (IG). The studied variables that could interfere were: age, gender, comorbidities, diagnosis of Crohn’s disease, previous abdominal surgery, inadequate preparation to compare the groups reached and did not reach the cecum.

**Results:**

Of the 939 patients included 879 (93.3%) reached the cecum (CG) and 63 (6.7%) IG no. The IG was composed of 29 (46.0%) men and 34 (54.0%) women with a mean age of 49.7 years; comorbidities this group accounted for 46% of which 15.9% was Crohn’s disease, previous abdominal surgery 22.2% and 17.5% inadequate preparation.

**Conclusion:**

Factors associated with complete or incomplete outcome of the examination with capsule endoscopy were: associated comorbidities, Crohn’s disease, previous abdominal surgery and inadequate preparation.

## Graphical Abstract

[Fig f1a]

**Figure f1a:**
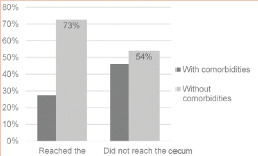
Results of CE between groups with or without associated comorbidities

## RESUMO

**Racional:**

O intestino delgado é segmento de maior dificuldade na visualização pelos métodos endoscópicos tradicionais. A necessidade de explorá-lo levou ao desenvolvimento da cápsula endoscópica. A porcentagem do exame completo por ela varia e falta identificar fatores que influenciam o exame completo e incompleto.

**Objetivo:**

Avaliar os fatores que interfiram na completude do estudo endoscópico pela cápsula.

**Método:**

Estudo prospectivo onde foram incluídos 939 pacientes divididos em dois grupos: grupo completo (GC) e grupo incompleto (GI). As variáveis analisadas que pudessem interferir foram: idade, gênero, comorbidades associadas, diagnóstico de doença de Crohn, operações abdominais prévias, preparo inadequado para comparar os grupos que atingiram e não atingiram o ceco.

**Resultados:**

Dos 939 pacientes incluídos 879 (93,3%) atingiram o ceco (GC) e 63 (6,7%) GI não. O GI era composto por 29 (46,0%) homens e 34 (54,0%) mulheres com idade média de 49,7 anos; as comorbidades deste grupo corresponderam a 46%, das quais 15,9% era doença de Crohn, 22,2% operação abdominal prévia e 17,5% preparo inadequado.

**Conclusão:**

Os fatores associados ao desfecho completo ou incompleto do exame com a cápsula endoscópica foram: comorbidades associadas, doença de Crohn, operação abdominal prévia e preparo inadequado.

## Graphical Abstract

[Fig f1as]

**Figure f1as:**
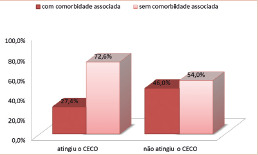
Resultados da CE entre os grupos com ou sem comorbidades associadas

## INTRODUCTION

The small bowel is the gastrointestinal tract site that has the greatest difficulty to be visualized by endoscopic traditional methods and, in it, endoscopy has limited access due to its length and the distance of accessible natural orifices^8^. The need to explore this relatively inaccessible segment led to the development of capsule endoscopy (CE)^12^. The study of the small intestine has improved significantly with the introduction of this device that has become standard method in the investigation of this segment^15^ ^,^ ^24^.

Currently, there are many indications to its use. Mainly, is indicated for gastrointestinal obscure bleeding, inflammatory bowel disease, celiac disease and small intestine cancer^12^ ^,^ ^15^ ^,^ ^24^.

Small bowel comprises approximately 75% of the obscure gastrointestinal bleeding, defined as digestive, persistent or recurrent in origin, and no recognized after colonoscopy^14^ ^,^ ^24^ ^,^ ^26^ and upper endoscopy. The obscure bleeding represents 3-5% of cases, is expensive and life-threatening^2^ ^,^ ^3^ ^,^ ^17^ ^,^ ^22^.

In obscure bleeding, or when other endoscopic examinations were inconclusive, further investigation with the CE is recommended^18^ ^,^ ^20^. It was approved in 2001 by the Food and Drug Administration (FDA)^12^ in the USA and allows visualization of the mucosa of the small intestine helping to establish the diagnosis^2,3,4,6,15,17,18,20,22.^

The percentage of complete examination by the CE varies and studies seek to identify factors that may influence lack of completeness that can limit its use. There are few publications on this topic in the international literature.

The objective of this study was to evaluate the factors that prevented the completion of the endoscopic study of the small intestine by the capsule.

## METHODS

This study was approved by the Research Ethics Committee of the Mackenzie Evangelical Faculty of Paraná, Curitiba, PR, Brazil and all patients signed informed consent prior to the examination. The design was prospective and observational.

Were included 939 patients who underwent CE in Gastrointestinal Endoscopy Department of the July 9 Hospital, São Paulo, SP, Brazil. The following variables were analyzed: gender, age, comorbidities, presence of Crohn’s disease, previous abdominal surgery, the inadequate preparation and the reach of the CE to the cecum in the test recording time. The life span of the batteries to keep the recording is about 8 h. The exam preparation was considered inadequate in the presence of residues or foodborne stasis which interfered with the proper mucosal evaluation. Data were recorded on a prospective spreadsheet. The capsules used were Mirocam^®^ Given^®^ and MA2 and SB2. All examinations were evaluated by two of the authors of this paper (PBP and TFS).

The preparation was the same for all patients, which consisted on the suspension of ferrous sulfate three days before the exam, and in the previous day, fed only with soft diet without waste or with clear liquids, administration of four bisacodyl tablets after lunch, intake of 1000 ml of water at 21 h with 100 drops of dimethicone, fasting of 10 h and 1 h before the procedure another ingestion of 1000 ml of water with 100 drops of dimethicone.

The independent variable was the capability of capsule endoscopy to reach the cecum. The images were captured by a portable recorder set at special abdominal belt for 8 h and transferred to the computer that processed the film with the help of specific software.

The patients were divided into two groups: complete study (CG) and incomplete (IG), which have or have not reached the cecum in the recording time.

Data were entered in Excel 2010 spreadsheets for Windows and statistical analyzes used the R version 3.0.2 program.

### Statistical analysis

It was made in a descriptive way through the mean, median, minimum and maximum values, standard deviation, absolute and relative frequencies (percentage), and graphics-dimensional dispersion and bars. The inferential analyzes performed in order to confirm or refute evidence found in the descriptive analysis were Chi-square or exact Fisher test^1^ when comparing the groups that reached or did not reach the cecum during recording time, by gender, presence of associated comorbidities, Crohn’s disease, previous abdominal surgery and inadequate preparation. Mann-Whitney^19^ was used when comparing the groups that reached and did not reach the cecum by age. The alpha level of significance was 5%.

## RESULTS

**FIGURE 1:**
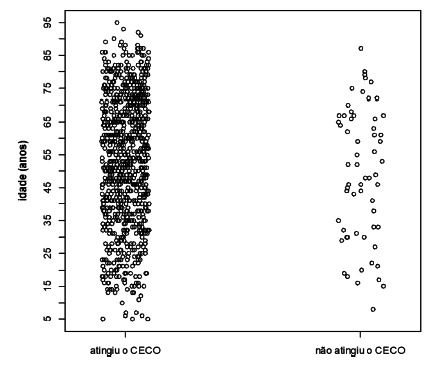
One-dimensional dispersion diagram of the age (years) of patients according to CE results

**FIGURE 2:**
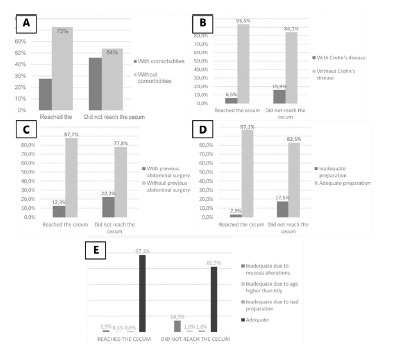
Results of CE between groups: A) with or without associated comorbidities; B) presence of Crohn’s disease; C) with previous abdominal operation; D) state of preparation; E) reasons why the preparation was inadequate, according to cases that reached or did not reach the cecum

Of the total sample of 939 patients, 462 (49.3%) were men and 476 (50.7%) women. The mean age was 53.2±19.6 years (5-95). In the CG, 433 (49.5%) were men and 442 (50.5%) women and in the IG 29 (46%) men and 34 (54%) women with p=0.596. The mean age in the CG was 53.5 years and in the IG 49.7 years with p=0.170 (Figure 1). From total, 268 (28.6%) patients had associated comorbidities, with Crohn’s disease in 67 (7.1%) and previous abdominal surgery 122 (13%, Figures 2A, 2B and 2C).

Among all cases, 879 (93.3%) reached the cecum and 63 (6.7%) did not (Figure 2D). Associated comorbidities were present in 239 (27.4%) in the CG and 29 (46%) in the IG (p=0.002). In CG 57 (6.5%) they had Crohn’s disease and in IG 10 (15.9%, p=0.011). Previous abdominal surgery had been performed in 108 (12.3%) in the CG and in 14 (22.2%, p=0.024) in the IG. Of the 63 patients who failed to reach the cecum, 11 (17.5%) had inadequate preparation (p<0.001). In these, the causes of inadequate preparation were subdivided (Figure 2E), which were attributed to: 1) changes in the mucosa (n=9, 81.8%); 2) age over 60 years (9.1%); and 3) only to inadequate preparation without other associated factors (9.1%,).

The group of 875 patients who had a complete CE study (CG) was formed by 433 (49.5%) men and 442 (50.5%) women. Their average age was 53.5±19.6 years (5-95). Associated comorbidity was present in 27.4% (n=239); Crohn’s disease in 6.5% (n=57) and previous abdominal operation 12.3% (n=108).

The inferential results confirmed the same gender (p=0.596) and age (p=0.170) profile, regardless of whether or not reached the cecum. The same behavior was not observed when comparing the presence of associated comorbidity (p=0.002), Crohn’s disease (p=0.011) and previous abdominal surgery (p=0.024); so, patients who have reached the cecum had fewer comorbidities, Crohn’s disease and previous abdominal surgery.

## DISCUSSION

Several authors have studied the complete transit of the CE achieving different percentages, but very close. According to Rahmi Gabriel et al.^16^ in long-term multicenter prospective study, the complete transit of the CE through the small intestine was achieved in 91.8%; Vlachogiannakos et al.^22^ showed 87%; and Hoedemaker et al.^9^ 81.8%. In a review involving 22,840 procedures, the percentage was 85.4%^11^. In the present study the full evaluation was possible in 93.3%, showing higher rate compared to most studies. Authors report incomplete rates ranging from 0-50%, which in these cases is unfavorable to the method for potential diagnosis loss^25^.

Retrospective studies have identified factors that may be predictive of incomplete examination; among them are mentioned bedridden patients, previous abdominal surgery and bowel inadequate preparation^25^. In this study, it was demonstrated that gender and age were not significant factors for both groups, which is also shown by other authors^13^ ^,^ ^21^ ^,^ ^23^ ^,^ ^25^.

The variables analyzed here - comorbidities, diagnosis of Crohn’s disease, previous abdominal surgery and inadequate preparation - were factors that showed be impeditive to complete examination.

Associated comorbidity (p=0.002) had significant difference between the two groups, which is corroborated by studies that add chronic diseases in hospital^5^ ^,^ ^13^ ^,^ ^25^ as a factor that contributes to the incomplete study.

Chronic diseases are associated with prolonged CE retention in the stomach due to longer gastric emptying time, which results in inadequate time for the complete evaluation of the small intestine, since the battery life is about 8 h^5,7,21^ .

Regarding Crohn’s disease, it is considered a significant factor for incomplete study (p=0.011). This has proven to be an important risk factor for the retention of CE^7^ ^,^ ^10^. Its diagnosis requires a combination of clinical, endoscopic and histological findings. Most image studies offer low sensitivity for early identification of the disease and, furthermore, upper gastrointestinal endoscopy and colonoscopy does not allow complete examination of the bowel. 

The CE has a higher sensitivity in the identification of mucosal changes; therefore, it has valuable role in bowel evaluation as a whole, and especially in those with known or suspected Crohn´s ^10^.

Studies associated previous history of intestinal obstruction as a predictive factor for the exam be incomplete^10^ ^,^ ^25^ circumstance more commonly found in patients with Crohn’s disease^7^ ^,^ ^10^.

The presence of previous abdominal surgery was significantly associated with incomplete study (p=0.024); this fact has been reported by other authors with very close ratios to herein^10^ ^,^ ^11^ ^,^ ^13^ ^,^ ^21^ ^,^ ^25^.

The inadequate preparation was significant in IG (p<0.001). Most IG patients (81.8%) presented mucosal changes such as edema, hyperemia, changes of the villi, fibrosis, stenosis, scar retractions, deformities, tacks, ulcers or diverticula. This suggests that the existence of chronic diseases, inflammatory bowel disease, tumor or obstruction can justify. Age as a possible factor to influence the proper preparation could be attributed to delayed gastric emptying; but, in this study, it was not the main cause (9.1%), as the same rate was found for the presence of residues or stasis, with no apparent cause.

The poor bowel preparation was also a significant factor for incomplete examination in other studies^9^ ^,^ ^10^ ^,^ ^13^ ^,^ ^21^ ^,^ ^25^.

## CONCLUSION

Factors associated with complete or incomplete outcome of the examination with capsule endoscopy were: associated comorbidities, Crohn’s disease, previous abdominal surgery and inadequate preparation.

## References

[1] Agresti A (1990). Categorical data analysis.

[2] Ali A, Santisi JM, Vargo J (2004). Video capsule endoscopy a voyage beyond the end of the
scope. Cleve Clin J Med.

[3] American Society for Gastrointestinal (2010). Endoscopy (ASGE). The role of endoscopy in the management of
obscure GI bleeding. Gastrointestinal Endoscopy.

[4] Banic M, Babic Z, Kujundzic M, Petricusic L, Urek-Crncevic M, Grgurevic I, Kardum D, Bokun T (2009). [Video capsule endoscopy--preliminary experience in university
hospital setting]. Acta Med Croatica.

[5] Ben-Soussan E, Savoye G, Antonietti M, Ramirez S, Lerebours E, Ducrotté P (2005). Factors that affect gastric passage of video
capsule. Gastrointest Endosc.

[6] Carol E (2009). Semrad Small Bowel enteroscopy Territory conquered, future
horizons. Disclosures. Curr Opin Gastroenterol.

[7] Cheon JH, Kim YS, Lee IS (2007). Can we predict spontaneous capsule passage after retention A
nationwide study to evaluate the incidence and clinical outcomes of capsule
retention. Endoscopy.

[8] da Costa RD, Kemp R, dos Santos JS, DAPD, Ardengh JC, Ribas-Filho JM, Ribas CAPM (2020). The role of conventional echoendoscopy (EUS) in therapeutic
decisions in patients with neuroendocrine gastrointestinal
tumors. ABCD Arq Bras Cir Dig.

[9] Hoedemaker RA, Westerhof J, Weersma RK, Koornstra JJ (2014). Non-small-bowel abnormalities identified during small bowel
capsule endoscopy. World J Gastroenterol.

[10] Kav T, Bayraktar Y (2009). Five years' experience with capsule endoscopy in a single
center. World J Gastroenterol.

[11] Kav T, Bayraktar Y (2009). Five years' experience with capsule endoscopy in a single
center. World J Gastroenterol.

[12] Mishkin DS, Chuttani R, Croffie J, DiSario J, Liu J (2006). ASGE Technology Status Evaluation Report: wireless capsule
endoscopy. Gastrointestinal Endoscopy.

[13] Lee Mitchell M (2010). Factors associated with incomplete small bowel capsule endoscopy
studies. World J Gastroenterol.

[14] Ohmiya N1, Nakagawa Y, Nagasaka M, Tahara T, Shibata T, Nakamura M, Hirooka Y, Goto H, Hirata I (2015). Obscure gastrointestinal bleeding diagnosis and
treatment. Dig Endosc.

[15] Pennazio M, Spada C, Eliakim R (2015). Small-bowel capsule endoscopy and device-assisted enteroscopy for
diagnosis and treatment of small bowel disorders European Society of
Gastrointestinal Endoscopy (ESGE) Clinical Guideline. Endoscopy.

[16] Gabriel Rahmi (2014). Long-term follow-up of patients undergoing capsule and
double-balloon enteroscopy for identification and treatment of small-bowel
vascular lesions: a prospective, multicenter study. Endoscopy.

[17] Segarajasingam DS, Hanley SC, Barkun AN, Waschke KA, Burtin P, Parent J, Mayrand S, Fallone CA, Jobin G, Seidman EG, Martel M (2015). Randomized controlled trial comparing outcomes of video capsule
endoscopy with push enteroscopy in obscure gastrointestinal
bleeding. Can J Gastroenterol Hepatol.

[18] Koh Seong-Joon, Im Jong Pil, Kim Ji Won, Kim Byeong Gwan, Lee Kook Lae, Kim Sang Gyun, Kim Joo Sung, Jung Hyun Chae (2013). Long-term outcome in patients with obscure gastrointestinal
bleeding after negative capsule endoscopy. World J Gastroenterol.

[19] SIEGEL S (2006). Estatística não-paramétrica para ciências do comportamento.

[20] Tan W, Ge ZZ, Gao YJ, Li XB, Dai J, Fu SW, Zhang Y, Xue HB, Zhao YJ (2015). Long-term outcome in patients with obscure gastrointestinal
bleeding after capsule endoscopy. J Dig Dis.

[21] Triantafyllou K, Kalantzis C, Papadopoulos AA, Apostolopoulos P, Rokkas T, Kalantzis N, Ladas SD (2007). Video-capsule endoscopy gastric and small bowel transit time and
completeness of the examination in patients with diabetes
mellitus. Dig Liver Dis.

[22] Vlachogiannakos J, Papaxoinis K, Viazis N, Kegioglou A, Binas I, Karamanolis D, Ladas SD (2011). Bleeding lesions within reach of conventional endoscopy in
capsule endoscopy examinations for obscure gastrointestinal bleeding is
repeating endoscopy economically feasible?. Dig Dis Sci.

[23] W (2005). Complete small-bowel transit in patients undergoing capsule
endoscopy: determining factors and improvement with
metoclopramide. Gastrointest Endosc.

[24] Westerhof J, Koornstra JJ, Weersma RK (2008). Capsule endoscopy a review from the clinician's
perspectives. Minerva Gastroenterol Dietol.

[25] Westerhof J, Weersma RK, Koornstra JJ (2009). Risk factors for incomplete small-bowel capsule
endoscopy. Gastrointest Endosc.

[26] Min Yang Won, Kim Jin Su, Jeon Seong Woo (2014). Long-term outcome of capsule endoscopy in obscure
gastrointestinal bleeding a nationwide analysis. Endoscopy.

